# The effects of the implementation order of implicit and explicit learning methods on learners' task enjoyment and motor skill acquisition

**DOI:** 10.3389/fspor.2025.1605959

**Published:** 2025-06-19

**Authors:** Yuki Matsuura, Hiroki Matsuoka, Yoshifumi Isa, Yosuke Sakairi

**Affiliations:** ^1^ Department of Health and Physical Education, Cooperative Faculty of Education, Utsunomiya University, Utsunomiya, Japan; ^2^ Department of Health Informatics, Faculty of Healthcare Management, Niigata University of Health and Welfare, Niigata, Japan; ^3^ Department of Health and Physical Education Teacher's Consultant, Okinawa Prefectural Education Center, Okinawa, Japan; ^4^ Department of Psychology and Education, Faculty of Education, Tokoha University, Shizuoka, Japan

**Keywords:** motor learning, learning method, college physical education, apparatus gymnastics, information and communication technology

## Abstract

**Purpose:**

This study aimed to explore the effect of the order of two learning methods (one based on implicit and another on explicit learning) on students' enjoyment and ability to acquire motor skills in gymnastics. Apparatus gymnastics courses for pre-service teachers were analyzed using information and communication technology.

**Methods:**

The participants were 21 pre-service teachers in Japan. They were divided into two groups with equal skill levels, with the order of learning method alternating between the groups. Seven lessons were conducted in total. Changes in enjoyment of learning mat exercises, physical sensation during practice, and self-evaluation of skill progress were examined before and after class.

**Results:**

In implicit learning, the learners enjoyed activities without concern about the presence of others; however, their self-evaluation of skill progress was lower than that in explicit learning. In explicit learning, learners enjoyed activities less than in implicit learning; however, their self-evaluation of skill progress was higher than that in implicit learning, and they tried to perform tasks with higher-level skills. This was possibly because learners experienced enjoyment without concern about the presence of others implicit learning, followed by the opportunity to improve their self-evaluation and attempt higher-level tasks in explicit learning.

**Conclusion:**

The findings suggest optimal instructional strategies should implement implicit learning to foster enjoyment and sensory-motor exploration, followed by explicit learning to enhance progress self-evaluation and promote performance of advanced skill challenges.

## 1 Introduction

Apparatus gymnastics^1^ is a compulsory component of physical education curricula in Japanese elementary and junior high schools (1, 2). Consequently, the majority of teacher-training curricula in Japanese universities designate it a mandatory subject in physical education practicum courses to obtain a teaching license. However, a survey of teachers conducted in Japanese public junior high schools revealed that apparatus gymnastics was the most challenging discipline for health and physical education teachers to instruct (3). Similarly, a study reported that approximately 70% of elementary school teachers experienced significant difficulties in its instruction (4). These findings underscore the need to develop instructional materials that can effectively support the teaching of apparatus gymnastics. Furthermore, many junior high and elementary school teachers, as well as university students enrolled in teacher-training programs, emphasize the importance of instructors possessing demonstrative competencies in apparatus gymnastics instruction (5). Thus, university students in teacher-training programs must cultivate the ability to proficiently demonstrate gymnastic classes.

To ensure that all learners experience intrinsic satisfaction with their skill acquisition in apparatus gymnastics education, tasks and objectives that align with learners' individual proficiency levels and practice methodologies tailored to specific learning challenges must be designed (6). Furthermore, traditional pedagogical approaches should be effectively integrated with information and communication technology (ICT) in a complementary manner to achieve these objectives (1, 2, 7).

Therefore, we developed ICT teaching materials that enable each learner to learn the skills of their choice at their own pace. Two distinct instructional approaches have been implemented: kinesthetic-experiential learning (KEL), characterized by implicit learning, and model-mastery learning (MML), characterized by explicit learning. Both methods have been examined in previous studies (8, 9). KEL is an implicit learning method that: (1) guides learners to focus on the kinesthetic cues most strongly associated with successful movement outcomes; (2) provides opportunities to explore a range of kinesthetic experiences; and (3) facilitates the implicit acquisition of the relationship between bodily sensations and the corresponding movement performance (9). MML is an explicit learning approach that offers step-by-step guidance and specific techniques for achieving optimal body control, drawing from multiple instructional sources. Matsuura et al. (10) conducted mat exercise classes for university students using this ICT material, allowing students to autonomously select their preferred learning method. As a result, the findings demonstrated that students were able to master the technique and engage in practice aligned with their abilities, regardless of their awareness of their motor proficiency and learning style.

Implicit learning has been advocated in the coaching literature because of its reported effectiveness in motor skill acquisition among athletes (11) and its lower susceptibility to performance deterioration under psychological pressure compared to explicit learning (12). However, the available implicit learning methods are problematic in that they are impractical (13). Accordingly, the ICT-based teaching material may help address this challenge. In a comparative study of implicit and explicit learning in gymnastics, both groups demonstrated similar improvements during learning; however, implicit learning proved more effective for long-term retention (14). Although many studies recommended implicit learning, as noted above, a meta-analysis by Kal et al. (15) reported minimal differences between the two approaches in promoting movement automatization. Furthermore, previous research has reported that the psychological and motor skill characteristics of these learning methods differ (9, 16). KEL (implicit) has been found to be more effective than MML (explicit) in enhancing learners' enjoyment of the content and intrinsic motivation, fostering a greater diversity of movements during the learning process. In contrast, while MML (explicit) is perceived as less enjoyable than KEL (implicit), it is more effective in improving technical performance (9, 16).

Thus, most previous research on implicit and explicit motor learning has examined these two as contrasting approaches (e.g., 14, 15, 17). However, it has been argued that the process of human skill acquisition often involves both implicit and explicit processes and therefore includes interactions between them (18). Considering the effects reported in previous studies (9, 16), it can be argued that there is value in examining the effective sequence of implementation that leverages the distinctive characteristics of both learning methods.

In cognitive skill learning, an integrated model that considers both implicit and explicit learning processes has been proposed (19). In the model, a bottom-up approach (first learning implicit knowledge and then explicit knowledge based on implicit knowledge) is adopted for low-level skill learning (18). However, this research focuses on cognitive skills, and it remains unclear whether the findings are applicable to practical motor learning. Focusing on the psychological effects of the implementation sequence, initiating learning with KEL, which possesses characteristics of implicit learning, may be effective for subsequent learning. The reason for this is that KEL can enhance learners’ sense of enjoyment toward the activity and increase motivation early in learning (9, 16). Another reason is that the level of physical enjoyment mediates the effectiveness of physical activity interventions on learners' participation in learning activities (20). Previous studies (9, 16) have reported enjoyment in terms of the degree of flow state (21). The flow state, widely thought to provide an optimal learning environment (22), is regarded as a crucial factor in the learning process, with research indicating that it has a significant positive influence on learning outcomes (23). Therefore, as evaluating the degree of flow state provides a critical perspective for understanding and enhancing the learning experience, this study also examines the degree of learners flow state.

Similarly, when focusing on motor skills, conducting KEL (implicit) followed by MML (explicit) is considered to align well with the natural variability that occurs during the learning process. Specifically, the progression of motor proficiency and variability follows a U-shaped curve (variability is high at the beginning of learning, decreases as learning progresses, and then increases again at more advanced stages of skill acquisition) (24), and learning aligned with this variability pattern is considered a viable strategy. It has also been suggested that introducing high variability early in the skill acquisition process supports mastery of the skill, and that the timing of this variability introduction is critical for effective learning (25). Based on these considerations, determining the optimal sequence of these learning methods in practical motor learning situations is essential for maximizing the benefits of both approaches—enhancing the sense of enjoyment toward the activity and promoting skill acquisition. Therefore, this study aimed to explore the effects of the sequence of learning method implementation on both enjoyment of the activity and motor skill acquisition. The order of implementation was systematically controlled by utilizing the ICT teaching material we developed, allowing learners to experience both methods.

## 2 Method

### 2.1 Participants

Twenty-one pre-service teachers instructing apparatus gymnastics classes (16 men and 5 women; mean age = 19.7 years, SD = 0.71) participated in this study. The participants' only experience with apparatus gymnastics was through physical education classes, and none of the students specialized in apparatus gymnastics. The participants were randomly divided into two groups (12 in the KEL-antecedent group and nine in the MML-antecedent group) to ensure that their skill levels in instrumental exercise were similar, and all instructional sessions were delivered by an instructor specializing in apparatus gymnastics. Skill level was assessed during the first class by having students perform basic techniques and exercises, with the instructor evaluating their performance. Although all the participants attended classes as part of their regular coursework, ethical considerations were meticulously observed. Specifically, participation in the study was at the voluntary discretion of the participants, in regard to the use of surveys before and after classes and access to learning records in the ICT teaching materials for research purposes. The participants were explicitly informed that their participation was entirely voluntary, and only those who provided informed consent were included in the study.

### 2.2 Contents and learning methods

**Figure 1:**
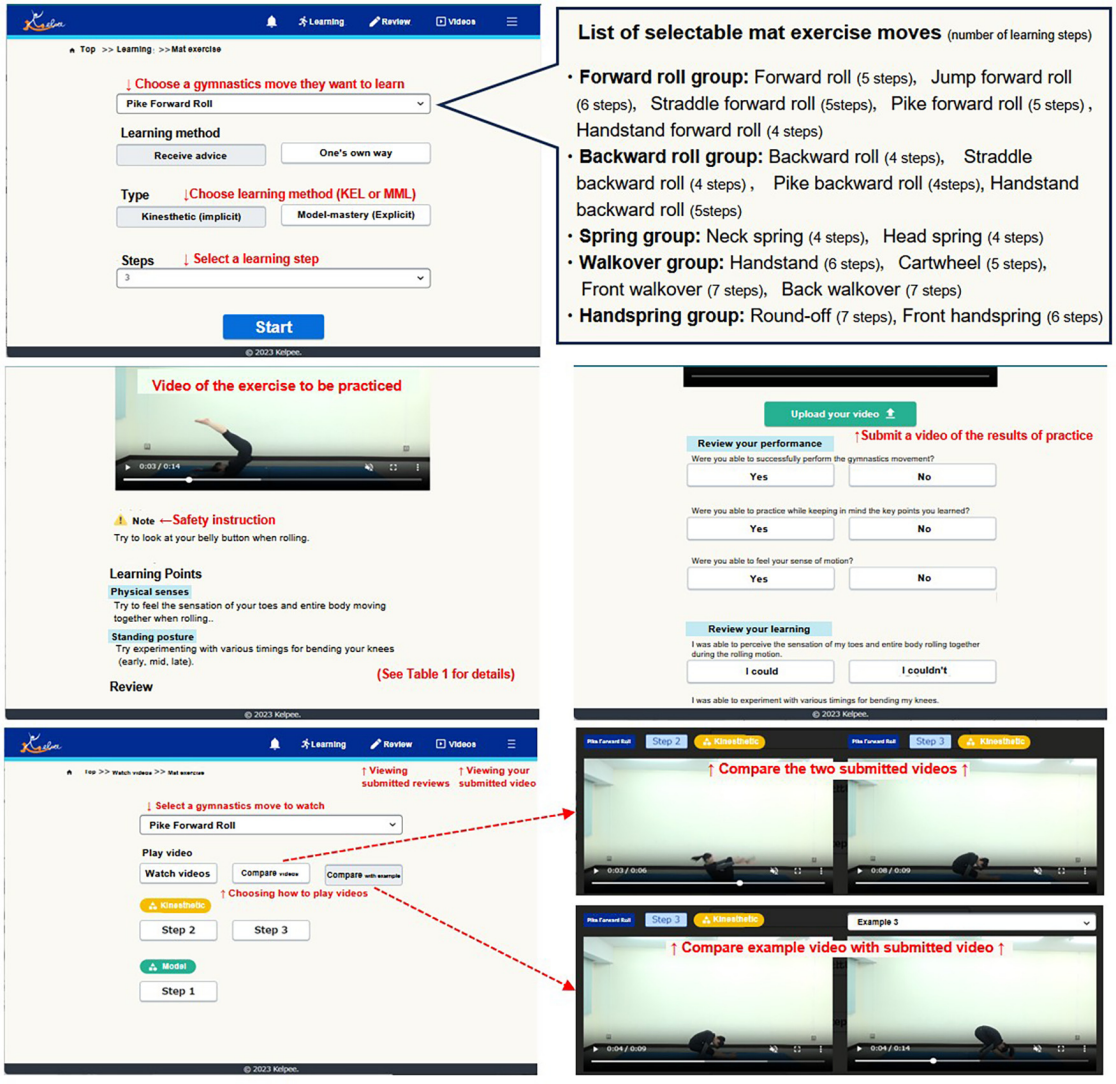
A part of the screen used by learners for the ICT teaching materials used in this study. The learners learn the skills using the ICT teaching materials that we developed. This ICT teaching material includes two distinct instructional approaches that have been implemented: kinesthetic-experiential learning (KEL) based on implicit learning method and model-mastery learning (MML) based on explicit learning method. The specific differences in learning methods are shown in Table 1.

The learning method using ICT teaching materials in this study was designed to facilitate step-by-step learning through a combination of KEL and MML, as described by Matsuura et al. (9, 16). KEL is an implicit learning method that (1) directs the learner's attention to the kinesthetic sensation that is most predictive of movement outcomes, (2) provides opportunities to experience a variety of kinesthetic sensations, and (3) enables the learner to implicitly learn the relationship between these sensations and their movement outcome, thereby mastering the skill (9). Accordingly, the instructional framework for KEL was designed to enable learners to independently discover effective techniques for mat exercises through diverse experiential learning opportunities. In contrast, MML is an explicit learning method that presents methods and tips for acquiring ideal body control in stages based on multiple instruction manuals. Consequently, MML followed a structured and explicit approach, and the content was designed based on multiple instructional books on mat exercises (26–28). The distinctions between the instructional phases and content are presented in Table 1, with pike forward roll provided as a representative example. Gymnastic skills targeted in the ICT teaching materials are aligned with those outlined in the course of study (1, 2). Learners could select a specific gymnastic skill from the provided list, review instructional videos and textual guidance corresponding to each learning level, and commence practice at their chosen proficiency stage (hereafter referred to as the “learning step”). Figure 1 presents some of the screens used by the learners.

**Table 1:** Comparison of instructional content for kinesthetic-experiential learning (implicit) and model-mastery learning (explicit) (steps 1, 3, and 5 of pike forward roll).

Steps	List	KEL		MML	
1	Practice content	Forward roll emphasizing knee extension		Forward roll emphasizing knee extension	
	Safety instruction^a^		Try to look at your belly button when rolling.		Try to look at your belly button when rolling.
	Learning points	Physical senses	Try to feel the sensation of your toes and entire body moving together when rolling.	Awareness of feet	When the back of your head touches the mat, extend your knees and stretch your legs forward.
		Standing posture	Try experimenting with various timings for bending your knees (early, mid, late).	Standing posture	Just before standing up, quickly bend your legs and stand up using momentum.
	Checklist^b^	Physical senses	I was able to perceive the sensation of my toes and entire body rolling together during the rolling motion.	Awareness of feet	When the back of my head touched the mat, I was able to extend my knees and stretch my legs forward.
		Standing posture	I was able to experiment with various timings for bending my knees.	Standing posture	Just before standing up, I was able to bend my legs and stand up using momentum.
2		Omitted			
3	Practice content	Lifting hips from a rolling motion with extended knees		Lifting hips from a rolling motion with extended knees	
	Learning points	Physical senses	Try to feel the sensation of your toes and center of gravity (around the hips and buttocks) moving together when rolling.	Awareness of feet	Roll backward with your knees extended, widen the angle of your hips, and bend forward while forcefully stretching your legs forward.
		Pressing wit your hands	Try experimenting with various timings for the pike and pressing with your hands (before, during, and after the pike).	Pressing with your hands	While rolling, press your hands firmly against the mat and lift your hips.
4		Omitted			
5	Practice content	Pike forward roll		Pike forward roll	
	Learning points	Physical senses	Try to perceive the relationship between your toes and center of gravity while rolling.	Awareness of feet	Roll with the back of your head on the mat and extend your knees as soon as your feet leave the mat.
		Awareness of feet	Always place the back of your head on the mat before rotating. Find the timing that works best for you to swing your legs downward and to press your hands against the mat and bend forward, as well as the best direction for your upper body when standing up. Then, stand up with your knees fully extended.	Pressing with your hands	As soon as your feet touch the mat, place your hands on your thighs and bend your upper body forward.
		Pressing with your hands		Timing for pike	
		Timing for pike		Standing posture	While bending forward, push off the mat strongly with your hands as you rotate and stand up smoothly.
		Standing posture			

KEL, kinesthetic-experiential learning; MML, model-mastery learning.

^a^ Guidance on safety aspects during execution and notes for the assistant are described for each technique.

^b^ The checklist corresponds to the learning content for each step. Therefore, checklists except those for Step 1 have been omitted.

Table 1 presents the specific instructional content for each learning method used in this study, using the Pike forward roll as an example. The instructional contents were determined through discussions among three experts—a university faculty member specializing in gymnastics coaching, a university faculty member specializing in sports psychology, and a researcher specializing in gymnastics. The content shown in Table 1 is displayed within the application illustrated in Figure 1, alongside a reference video. During the mat exercise class, each student used a tablet device to practice the mat exercise technique of their choice, following the designated learning method via the application. The instructor supervised the students, providing safety guidance as needed and responding to their questions. To ensure consistent instruction across all participants, only the specific content outlined in Table 1 was provided.

### 2.3 Measurement

#### 2.3.1 Sport flow

The Sport Flow Scale (SFS; 29) was used to assess the degree of enjoyment experienced during mat exercise practice similar to previous research (16). This scale comprises six items corresponding to six sub-factors: (1) merging of action and awareness, (2) concentration on the task at hand, (3) sense of control, (4) loss of self-consciousness, (5) transformation of time, and (6) autotelic experience. Responses were rated on an 11-point Likert scale. Responses were obtained via an online form.

#### 2.3.2 Awareness of bodily sensations

To assess the participants' awareness of bodily sensations during mat exercise skills practice, they were asked to respond to a five-item questionnaire, ranging from “awareness of concrete body movements” (1) to “awareness of abstract bodily sensations” (5), as well as from “awareness of partial body movements, such as hand movements and foot positioning” (1) to “awareness of whole-body movement while performing gymnastic techniques” (5). Responses were obtained via an online form.

#### 2.3.3 Number of learning steps and skill evaluation

To assess students' self-evaluation of progress in mat exercise skills, they were asked to evaluate their own advancement before and after the class using a five-point scale, ranging from “no progress at all” (1) to “significant progress” (5). Responses were obtained via an online form. Additionally, for an objective assessment of mat exercise proficiency, the instructor evaluated the skills practiced using the ICT teaching material during the seventh class based on the following grading criteria: 100 points (excellent), 70 points (good), 40 points (average), and 10 points (poor). In this study, each student's evaluation score was derived from the average score of all assessed techniques. Furthermore, the number of learning steps completed and difficulty level of the learning steps attempted by each student in each lesson were extracted from the data recorded in the ICT teaching materials.

#### 2.3.4 Procedure

**Figure 2:**
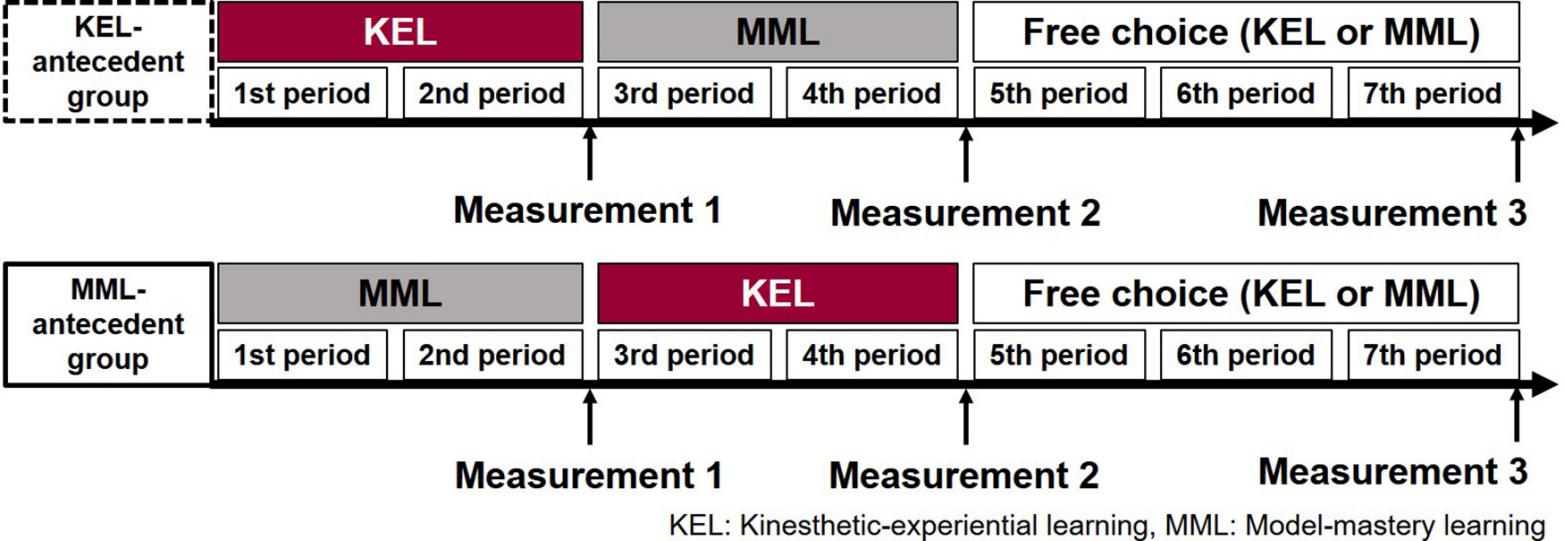
Experimental protocol. Figure shows the research protocol. In the KEL-antecedent group, participants engaged in KEL (implicit) during the first two sessions, followed by MML (explicit) in the third and fourth sessions. Sessions five through seven allowed learners to freely select their preferred learning method. The MML-antecedent group learned in the reverse order. Both groups completed questionnaires measuring SFS, self-assessed skill improvement, and bodily awareness during the mat exercise after sessions two (Measurement 1), four (Measurement 2), and seven (Measurement 3). An instructor-administered skill evaluation was also conducted in the seventh session.

The participants engaged in seven instructional sessions that focused on mat exercise skill development using ICT teaching materials. In the KEL-antecedent group, the instructional sequence consisted of KEL for the first two sessions, MML for the third and fourth sessions, and the fifth through seventh practice sessions conducted with a free choice of learning method. Conversely, in the MML-antecedent group, MML was implemented in the first two sessions, KEL in the third and fourth sessions, and the fifth through seventh sessions conducted with a free choice of learning method. Both groups completed questionnaires assessing SFS, self-evaluation of skill progression, and awareness of bodily sensations during the mat exercise after the second (Measurement 1), fourth (Measurement 2), and seventh (Measurement 3) sessions. Responses were obtained via an online form. In addition, an instructor-led skill assessment was conducted during the seventh session. The research protocol is illustrated in Figure 2.

### 2.4 Analysis

Two-way analysis of variance (ANOVA) was conducted on the scores of each measure in a group (KEL-antecedent or MML-antecedent) × measurement time period between-subjects design. Multiple comparison tests were performed when significant interactions were observed. The Bonferroni correction was used to adjust for multiple comparisons. For the scores obtained in Measurement 3, a Mann–Whitney *U*-test was conducted based on the learning method selected by each participant. For teachers' practical test scores, a Mann–Whitney *U*-test was conducted using the KEL- and MML-antecedent groups. In all the statistical analyses, the significance level was set at *p* < .05, while a marginal trend was considered at *p* < .10. Statistical analyses were conducted using the IBM SPSS Statistics 28. No statistical sample size calculations were performed. However, to determine the statistical power, based on the sample size of this study (*n* = 21), a *post hoc* analysis was conducted using G*power 3.1 for a two-way mixed ANOVA designs and *U*-test with a medium effect size (*η_g_*^2^ = 0.25, *d* = .05) and a significance level of *p* < .05. The *post hoc* powers were 0.752 and 0.269, respectively.

## 3 Results

### 3.1 Sport flow

**Figure 3:**
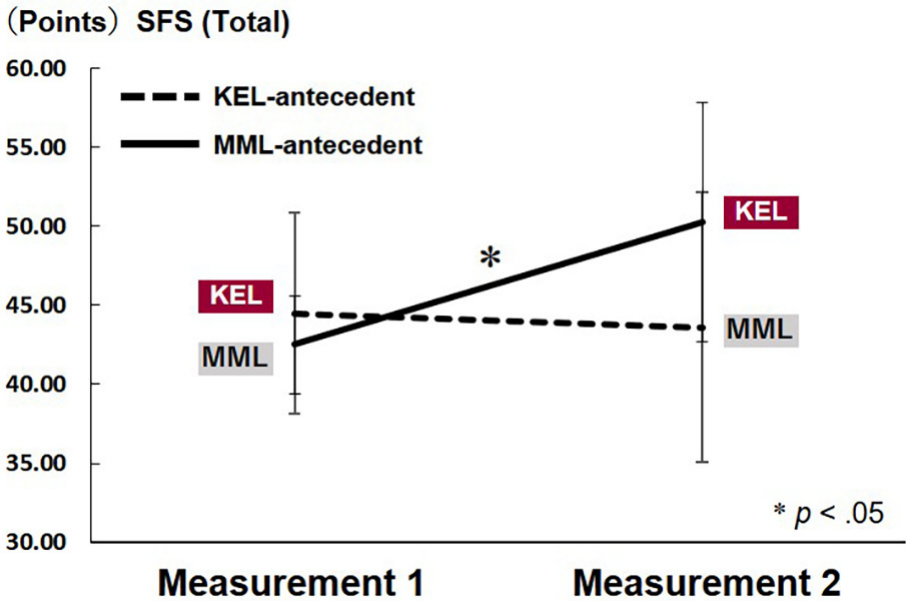
Comparison of SFS scores between the KEL- and MML-antecedent groups. Figure shows a comparison of the enjoyment (flow) scores for different learning methods. In the MML-antecedent group, the results revealed that implementing KEL after MML significantly increased the flow score, an indicator of enjoyment, when KEL was performed.

A significant interaction effect was observed for the total SFS scores (*F*_(1,10)_ = 5.091, *p* = .048). Multiple comparison tests revealed a significant increase in SFS scores from Measurement 1 (MML implementation) to Measurement 2 (KEL implementation) in the MML-antecedent group (*p* = .032) (Figure 3). Significant interaction effects were also identified for the subfactors “merging of action and awareness” (*F*_(1,10)_ = 7.511, *p* = .021) and “autotelic experience” (*F*_(1,10)_ = 6.275, *p* = .031). Multiple comparison tests indicated that, in Measurement 2, where the learning methods were switched, the scores for “merging of action and awareness” were significantly higher in the MML-antecedent group (KEL implementation) compared to the KEL-antecedent group (MML implementation) (*p* = .003). Furthermore, from Measurement 1 to Measurement 2, the scores for merging of action and awareness tended to increase in the MML antecedent group (MML → KEL) (*p* = .066), whereas a decreasing trend was observed in the KEL antecedent group (KEL → MML) (*p* = .098). Regarding the autotelic experience scores, the MML-antecedent group (KEL implementation) tended to score higher than the KEL-antecedent group (MML implementation) in Measurement 2 (*p* = .060). Additionally, a significant increase in autotelic experience scores was observed in the MML-antecedent group from Measurement 1 (MML implementation) to Measurement 2 (KEL implementation) (*p* = .044). For the remaining subfactors, neither the interaction effects nor the main effects of group or time were statistically significant (Table 2).

**Table 2:** Comparison of SFS scores, awareness during practice, and skill evaluation scores between the KEL- and MML-antecedent groups.

Measurement item		KEL-antecedent *n* = 12^a^		MML-antecedent *n* = 8^a^		Main effects		
		Measurement		Measurement		Interaction	Time	Condition
		1 (KEL)	2 (MML)	1 (MML)	2 (KEL)			
		*M* (SD)	*M* (SD)	*M* (SD)	*M* (SD)	*F*	*F*	*F*
SFS	Merging of action and awareness	7.38 (1.30)	6.13 (1.46)	7.00 (0.82)	9.00 (0.00)	7.511*	0.400	8.333*
	Concentration on the task at hand	8.75 (1.28)	7.63 (1.41)	9.25 (0.96)	9.50 (0.58)	1.967	0.797	4.610^†^
	Sense of control	6.38 (2.20)	7.00 (1.31)	6.75 (2.99)	8.50 (1.73)	0.381	1.697	1.256
	Loss of self-consciousness	7.00 (3.25)	8.00 (1.41)	8.00 (2.83)	6.75 (4.72)	0.005	0.033	2.660
	Transformation of time	6.38 (3.02)	6.75 (3.24)	3.25 (3.77)	6.75 (2.22)	1.336	2.055	1.336
	Autotelic experience	8.63 (1.51)	8.13 (1.46)	8.25 (2.06)	9.75 (0.50)	6.275*	1.569	0.583
	Sport flow (total)	44.50 (6.39)	43.63 (8.53)	42.50 (3.11)	50.25 (7.59)	5.091*	3.235	0.355
Awareness of bodily sensations	Concrete—abstract	3.29 (1.10)	2.04 (1.23)	1.94 (1.05)	3.00 (1.44)	9.065**	0.060	0.254
	Part—whole	3.29 (1.15)	2.79 (1.45)	2.25 (1.13)	2.13 (1.03)	0.297	0.826	3.892^†^
Motor skills	Self-evaluation of skill improvement	3.04 (0.81)	3.46 (0.72)	2.81 (0.88)	2.00 (0.93)	4.159^†^	0.431	14.084***
	Learning steps	2.00 (0.87)	1.44 (0.53)	1.86 (1.46)	1.57 (0.79)	0.256	2.493	0.000
	Difficulty level of the learning steps	3.31 (0.88)	3.72 (1.28)	4.20 (0.75)	3.40 (1.52)	3.165^†^	0.309	0.386

KEL, kinesthetic-experiential learning; MML, model-mastery learning.

^a^ SFS: KEL-antecedent: *n* = 8, MML-antecedent: *n* =  4.

^†^ *p* < .10, *p < .05, **p < .01, ***p < .001.

### 3.2 Awareness of bodily sensations

**Figure 4:**
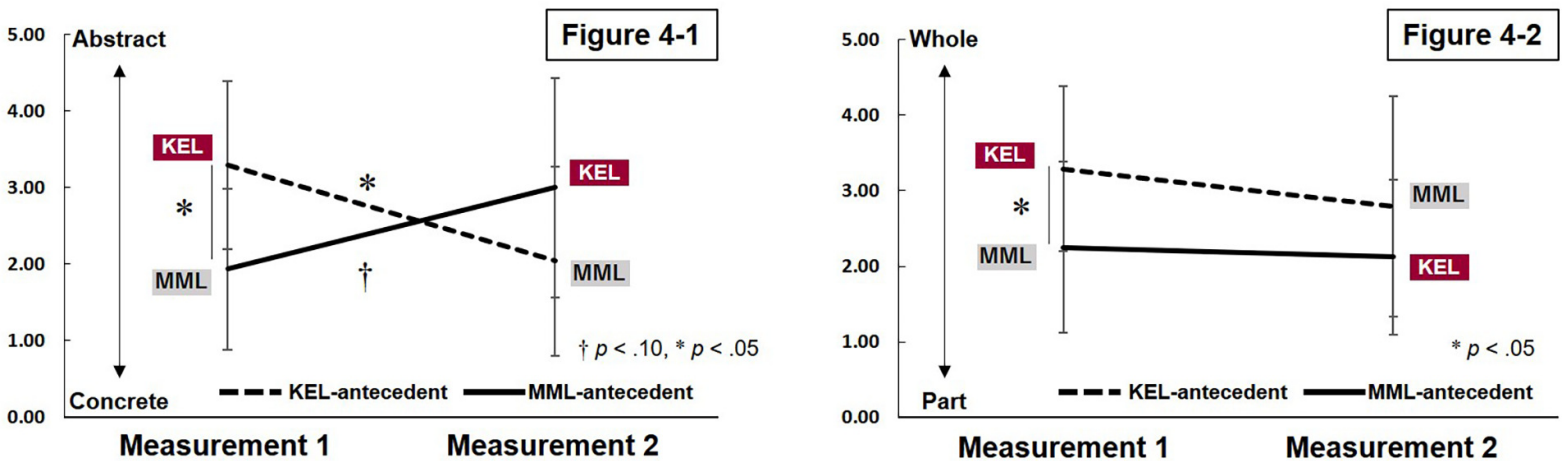
Comparison of awareness of bodily sensations during practice between the KEL- and MML-antecedent groups. Figure 4-1 shows a comparison of whether the participants were more attuned with “(1) Concrete awareness of how to move the body” to “(5) Abstract awareness of bodily sensations” while practicing mat exercise techniques. Participants exhibited increased awareness of abstract bodily sensations rather than concrete physical movements during KEL practice, whereas MML prompted greater attention to concrete movements over internal bodily sensations. Figure 4-2 shows a comparison of whether the participants were more aware of “(1) Partial movements” to “(5) Whole body movements” while practicing mat exercise techniques. Even when shifting learning methods, participants in the KEL-antecedent group generally retained their attention to whole-body movement, while those in the MML-antecedent group continued to emphasize movement in specific body parts.

A significant interaction effect was observed regarding participants' awareness during mat exercise skill practice, specifically on whether they focused on “awareness of concrete body movements” or “awareness of abstract bodily sensations” (*F*_(1,18)_ = 9.065, *p* = .008). As a result of multiple comparisons, in Measurement 1, the KEL-antecedent group (KEL implementation) exhibited greater awareness of abstract bodily sensations, whereas the MML-antecedent group (MML implementation) demonstrated a stronger focus on the concrete body movements (*p* = .013). In addition, in the KEL antecedent group, a significant change was observed from Measurement 1 (KEL implementation) to Measurement 2 (MML implementation) in the direction of greater awareness from abstract bodily sensations to concrete body movements (*p* = .019). In the MML antecedent group, a significant change was observed from Measurement 1 (MML implementation) to Measurement 2 (KEL implementation) in the direction of greater awareness from concrete body movements to abstract bodily sensations (*p* = .091) (Table 2 and Figure 4-1).

The main effects of interaction (*F*_(1,18)_ = 0.297, *p* = .592) and time period (*F*_(1,18)_ = 0.826, *p* = .376) on whether participants focused more on “partial movement” or “total body movement” during the mat exercise were not statistically significant. However, a marginal trend in antecedent learning was observed (*F*_(1,18)_ = 3.892, *p* = .064), indicating that the KEL-antecedent group tend to maintain a focus on “whole-body movement” awareness, while the MML-antecedent group maintained a focus on “partial movement” awareness (Table 2, Figure 4-2).

### 3.3 Number of learning steps and skill evaluation

**Figure 5:**
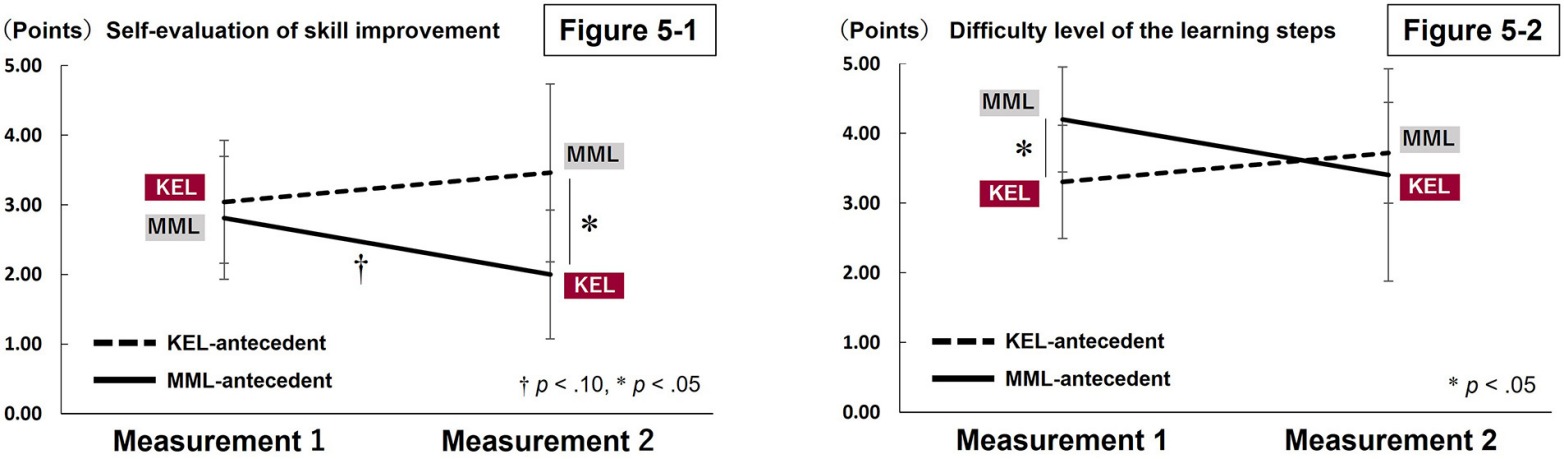
Comparison of self-evaluation of progress in mat exercise skills and difficulty level of the learning steps between the KEL- and MML-antecedent groups. Figure 5-1 compares the self-evaluation of progress in mat exercises, ranging from “no progress at all” (1) to “significant progress” (5). In the MML-antecedent group, self-assessed progress tended to decline when KEL was introduced following MML instruction. Figure 5-2 compares the difficulty level of the learning steps attempted by the learner. In the KEL-antecedent group, the difficulty level of the executed steps tended to be higher when MML was introduced following KEL instruction.

The instructor's skill evaluation scores revealed no statistically significant difference between the KEL- and MML-antecedent groups (*U* = 25.50, *p* = .864) (Table 3). A marginal interaction trend was observed in the self-evaluation of skill improvement (*F*_(1,18)_ = 4.159, *p* = .056). Multiple comparisons showed that in Measurement 2, where the learning methods were switched, the KEL antecedent group (MML implementation) had a significantly higher self-evaluation of skill improvement compared to the MML antecedent group (KEL implementation) (*p* = .001). Additionally, in the MML antecedent group, there was a tendency for self-evaluation of skill improvement to decrease from Measurement 1 (MML implementation) to Measurement 2 (KEL implementation) (*p* = .099) (Table 2 and Figure 5-1).

**Table 3:** Comparison of practical assessment scores between the KEL- and MML-antecedent groups by instructors.

Measurement item	KEL-antecedent *n* = 9		MML-antecedent *n* = 6		*U*	*p*
	Median (IQR)		Median (IQR)			
Instructor evaluation	70.00	(65.00–80.00)	70.00	(67.19–75.31)	25.50	0.85

KEL, kinesthetic-experiential learning; MML, model-mastery learning.

Neither interaction effect (*F*_(1,18)_ = 0.256, *p* = .620) nor the main effects of time period (*F*_(1,18)_ = 2.493, *p* = .137) and antecedent learning method (*F*_(1,18)_ = 0.000, *p* = .984) were statistically significant for the number of steps performed per lesson, indicating that there were no significant differences between the groups (Table 2). A marginal trend was observed in the interaction regarding difficulty level of the learning steps performed (*F*_(1,18)_ = 3.165, *p* = .097). Multiple comparison tests revealed that the MML antecedent group (MML implementation) tended to perform more difficult steps than the KEL antecedent group (KEL implementation) in Measurement 1 (*p* = .050) (Table 2 and Figure 5-2).

### 3.4 Comparison of learning methods

A comparison of the scores in Measurement 3, based on the learning methods freely chosen by the learners, revealed that the KEL-selected group demonstrated a significantly higher score for loss of self-consciousness, a sub-factor of the SFS, compared to the MML-selected group (*U* = 1.00, *p* = .004). Regarding awareness during mat exercise skill practice, the KEL-selected group focused on “abstract awareness of bodily sensations,” whereas the MML-selected group emphasized “concrete awareness of body movements” (*U* = 7.00, *p* = .032). No statistically significant differences were observed in the remaining measures (Table 4).

**Table 4:** Comparison of SFS scores, awareness during practice, and skill evaluation scores between the KEL- and MML-selected groups.

Measurement item		KEL-selected (Measurement 3) *n* = 10	MML-selected (Measurement 3) *n* = 4	*U*	*p*
		Median (IQR)	Median (IQR)		
SFS	Merging of action and awareness	7.00 (4.75–8.25)	7.50 (6.25–8.75)	25.000	0.539
	Concentration on the task at hand	8.00 (6.75–10.00)	8.50 (7.25–9.00)	19.000	0.945
	Sense of control	8.00 (6.00–9.00)	7.00 (3.25–7.75)	12.500	0.304
	Loss of self-consciousness	8.50 (7.00–9.25)	3.50 (2.25–5.50)	1.000	0.004***
	Transformation of time	6.50 (5.00–9.00)	7.50 (2.50–9.50)	22.000	0.839
	Autotelic experience	8.00 (6.75–10.00)	7.50 (5.50–8.75)	14.500	0.454
	Sport Flow (total)	44.00 (37.75–53.75)	42.50 (30.50–44.75)	14.000	0.454
Awareness of bodily sensations	Concrete—abstract	3.67 (2.00–4.00)	1.92 (1.08–3.13)	7.000	0.032*
	Part—whole	2.67 (1.92–4.08)	1.67 (1.08–3.25)	11.500	0.102
Motor skills	Self-evaluation of skill improvement	2.83 (2.67–3.38)	2.25 (1.38–3.75)	21.000	0.624
	Learning Steps	2.50 (1.75–6.25)	3.50 (2.25–4.75)	32.500	0.477
	Difficulty level of the learning steps	4.79 (4.38–5.00)	4.50 (3.81–5.00)	26.000	1.000

KEL, kinesthetic-experiential learning; MML, model-mastery learning.

*p < .05, ***p < .001.

## 4 Discussion

### 4.1 Psychological effects

In the MML-antecedent group, the results revealed that implementing KEL after MML significantly increased the flow score, an indicator of enjoyment, when KEL was performed. In terms of sub-factors, “merging of action and awareness,” which indicated the degree of spontaneous movement of the body, and “autotelic experience,” which indicated the sense of finding enjoyment in the activity itself, were significantly increased when KEL was implemented. Furthermore, the groups that chose KEL when given the freedom to select their learning method had significantly higher scores for “loss of self-consciousness,” indicating reduced concern about being observed by others. Regarding the self-evaluation of skill improvement, the MML-antecedent group showed a tendency for scores to decrease when KEL was implemented after MML. Regarding the difficulty of the steps attempted, there was a tendency for participants to attempt more difficult steps when performing MML.

KEL did not provide specific methods or techniques of movement; rather, it required learners to attempt various ways of performing the movements that corresponded to the tricks and experience different sensations to find the tricks on their own. In other words, learners were asked to explore ways of movement that they feel comfortable with, and this may have influenced the increase in the “merging of action and awareness” score. This indicates the degree of spontaneous movement of the body.

Furthermore, KEL did not prescribe a singular correct approach to movement. Instead, rigid value judgments regarding success or failure in performing exercises were inherently minimized by necessitating the exploration and experience of diverse movement patterns and bodily sensations. This principle aligns with the “freedom to fail” concept of gamification, a pedagogical approach that integrates game-like elements, such as interactive engagement in non-game contexts, to enhance learner motivation (30, 31). Research suggests that when instructors grant learners the “freedom to fail,” their engagement, participation, and overall learning outcomes are significantly enhanced (32). These factors may have contributed to the observed increase in autotelic experience and loss of self-consciousness during skill acquisition.

However, in the MML-antecedent group, self-evaluation of progress tended to be lower when KEL was administered after MML. This result may be attributed to the inherent nature of KEL, which, as noted above, did not provide explicitly defined movement solutions. In other words, the absence of a definitive “correct answer” may have created ambiguity regarding the extent of skill improvement. Additionally, the difficulty level of the executed steps tended to be higher when MML was implemented. This finding suggests that MML, which explicitly presented correct movement models and assessment criteria, may have enhanced participants' motivation to “successfully execute the technique” and “pursue higher levels of skill proficiency.”

### 4.2 Effects on motor skills

Although no significant differences were observed between the two learning methods in terms of the instructor's skill evaluation scores, it was evident that students’ awareness of bodily sensations during technique execution varied depending on the learning method. Specifically, participants demonstrated a heightened awareness of abstract bodily sensations rather than concrete body movements when practicing in KEL, whereas MML led to a greater focus on concrete body movements than on bodily sensations. This finding indicated that the participants adapted their cognitive focus in alignment with the instructional framework, as KEL emphasized sensory awareness and experiential learning, whereas MML provided explicit movement techniques and prescriptive strategies for skill execution.

Awareness of whole-body and partial-body movements may have been influenced by the preceding learning method. Following the transition in learning method, the KEL-antecedent group tended to sustain their awareness of whole-body movement, whereas the MML-antecedent group maintained its focus on partial body movement. This phenomenon may be attributed to the cognitive framework established during the initial skill acquisition. When participants in the KEL group learned a technique through abstract bodily sensations, they were more likely to carry this sensory-based approach over to subsequent skill acquisition. Conversely, when MML was first introduced, the explicit strategies and technical cues acquired in the initial session may have served as a cognitive foundation for subsequent learning experiences.

### 4.3 Order of learning method implementation

From a psychological perspective, implementing KEL in the early stages of learning is considered effective. This is because KEL allows for more enjoyable engagement in activities than MML and enhances intrinsic motivation (16). Conversely, when KEL was introduced at later stages of learning, the self-evaluation of progress tended to decline relative to MML, and the difficulty level of the tasks they tried also tended to decrease. Based on these findings, the following instructional sequence is considered effective. First, KEL is implemented to allow learners to experience the inherent enjoyment of the movement task and enhance their intrinsic motivation. The transition to MML helps learners recognize their progress and encourages them to take on slightly more challenging tasks.

For motor skills, highly variable learning methods such as KEL may be more effective in the long run in terms of learning effects if they are implemented first. First, various sensorimotor experiences, which constitute the core of KEL, support the diversity practice hypothesis (33) based on schema theory (34). This perspective posits that sensorimotor schema formation is facilitated under variable practice conditions where movement patterns are performed in diverse and adaptable ways. This was particularly true during the early stages of learning. Accumulating a variety of movement experiences in the early stages of learning effectively maps the possibilities of movement in a motor task onto the body (35). Furthermore, learners with greater variability in their movements for a specific motor task are more likely to acquire knowledge on how to adjust their movements, thereby accelerating the rate of motor learning (36). In addition, a U-shaped curve exists in the progression of motor proficiency and variability, where variability is high at the beginning of learning, decreases as learning progresses, and increases again at the more proficient stage (24). It has also been reported that children's motor learning is largely implicit (37). These findings collectively suggest that introducing a highly variable learning method, such as KEL, in the early stages of learning may be advantageous for fostering adaptability and motor schema development. Subsequently, as variability naturally declines with skill progression, implementing structured and technically explicit instructions, such as MML, may further refine movement execution and optimize skill acquisition.

### 4.4 Limitations of the study and future directions

One limitation of this study lies in the limited generalizability of its findings. Specifically, the study targeted participants enrolled in apparatus gymnastics classes, which allowed for more practical on-site findings. However, the sample size was not sufficient to avoid gender bias. Therefore, caution should be exercised when attempting to generalize these findings. Future studies should aim to accumulate more comprehensive evidence by considering individual characteristics and incorporating a more diverse sample in terms of gender, age, and exercise experience. Additionally, expanding the types of exercise tasks will contribute to the generalizability of the findings. Furthermore, increasing the sample size will also be essential for enhancing the reliability and validity of the findings.

Another limitation is that motor skill performance was assessed using only two perspectives: objective evaluation by the instructor and subjective evaluation by the learners. No kinematic analysis was conducted to determine the specific changes in motor skills. Furthermore, because of time constraints, this study did not examine how the order of instructional implementation affected motor skill development, highlighting the need for longer-term investigations. Future studies should explore the practical applicability of these findings in physical education and sports instruction by incorporating kinematic analyses, of motor skills, a wider range of motor tasks and learning periods, and more diverse participants group.

### 4.5 Practical recommendations

The results of this study suggest that, in motor skill learning, performing KEL (implicit learning) first and MML (explicit learning) later is effective psychologically (motivation, enjoyment, confidence) and technically (kinesthetic schema, technique refinement, U-shaped curve of variability associated with learning). Although further research is required to examine longer-term effects, the findings could be used as a practical framework for fostering learners' autonomy, increasing motivation, and promoting effective skill acquisition.

#### 4.5.1 Early learning stage: KEL (implicit learning)

- Guarantees “freedom to fail,” and enhances enjoyment and intrinsic motivation for learning tasks.
- Involves highly variable practice that emphasizes comprehensive sensation experiences, accumulates a wide variety of movement variations, which promotes the creation of a foundation for sensorimotor schema.

#### 4.5.2 Mid-stage learning: MML (explicit learning)

- Establishes clear goals, enabling learners to easily assess the degree of improvement.
- Encourages motivation to improve the perfection of techniques and engage with more difficult tasks.

## 5 Conclusion

This study investigated the effects of implementation sequence of KEL implicit learning and MML explicit learning on learners' task enjoyment and motor skill acquisition. The results revealed that KEL enabled learners to enjoy learning while experiencing a variety of motor sensations; however, it resulted in lower progress in self-evaluations and lower difficulty of tasks attempted than MML. These findings suggest that the optimal instructional strategy should implement KEL in the initial phase to foster enjoyment and sensory-motor exploration, followed by MML to enhance progress self-evaluation and motivate learners to attempt advanced skill challenges.

## Data Availability

The raw data supporting the conclusions of this article will be made available by the authors, without undue reservation.

## Footnotes

^1^Although both “Apparatus gymnastics” and “Artistic gymnastics” refer to the same sport, in Japan, they are distinguished by terminology. “Artistic gymnastics” refers to judged competitions that are held as official events at events such as the Olympics. “Apparatus gymnastics,” on the other hand, is one of the learning areas taught in school physical education classes. It has been adopted as teaching material for school physical education and has developed independently, and students are expected not only to learn new techniques but also to improve their ability to control their bodies while engaging in enjoyable activities (1, 2). In this study, we use the term “Apparatus gymnastics” because it focuses on gymnastics taught in school physical education.

## References

[1] MEXT. *The Government Curriculum Guidelines for Junior High Schools: Health and Physical Education*. Kyoto: Higashiyama (2017).

[2] MEXT. *The Government Curriculum Guidelines for Elementary Schools: Physical Education*. Tokyo: Toyokan (2017).

[3] Hasegawa K Akamitsu T Kurokawa T Mori H Hirata Y Ogura A. A research on systematic teaching in gymnastics classes in schools - through a questionnaire survey to elementary schools and junior high schools teachers-. Bull Int Pac Univ. (2018) 12:157–66. 10.24767/00000566

[4] Shimizu K Shiobara S Kaneko Y Sekiguchi A Takashima T Arai Y. Awareness about gymnastics education among elementary school teacher. Bull Gunma Univ Educ Pract. (2019) 36:107–16.

[5] Mizushima K. A study of teaching method of apparatus gymnastics. Bull Tokyo Gakugei Univ. (2004) 56:103–19.

[6] MEXT. School physical education practical instruction materials, volume 10: guide to apparatus gymnastics instruction (2016). Available at: https://www.mext.go.jp/component/a_menu/sports/detail/__icsFiles/afieldfile/2016/01/27/1356131_1.pdf (Accessed March 16, 2025).

[7] MEXT. Towards the realization of the GIGA school concept (2020). Available at: chrome-extension://efaidnbmnnnibpcajpcglclefindmkaj/https://www.mext.go.jp/content/20200625-mxt_syoto01-000003278_1.pdf (Accessed March 16, 2025).

[8] Matsuura Y Kokubu M Sakairi Y. Effects of versatile kinesthetic experiences on balance ability and interpersonal relationships. Psychol Rep. (2022) 125(2):1145–64. 10.1177/0033294120988133 33573502

[9] Matsuura Y Kokubu M Sakairi Y. Improvement of the ability to recover balance through versatile kinesthetic learning experiences. Front Sports Act Living. (2023) 4:975304. 10.3389/fspor.2022.975304 36733957 PMC9888364

[10] Matsuura Y Isa Y Sogabe T Matsuoka H. The effects of versatile kinesthetic learning experiences and ICT learning materials in mat exercises: an examination of the relationship between learner’s characteristics and learning behavior. The 74th Conference of Japan Society of Physical Education, Health and Sport Sciences (2023); ID: 3c401-12-05.

[11] Nijmeijer EM Brals FD Kempe M Elferink-Gemser MT Benjaminse A. How are athletes trained to move? A systematic review exploring the effects of implicit and explicit learning on biomechanics of sport-specific tasks. J Biomech. (2025) 184:112671. 10.1016/j.jbiomech.2025.112671 40209584

[12] Cabral DA Wilson AE Miller MW. The effect of implicit learning on motor performance under psychological pressure: a systematic review and meta-analysis. Sport Exerc Perform Psychol. (2022) 11(3):245–63. 10.1037/spy0000286

[13] Collins D MacPherson AC Bobrownicki R Carson HJ. An explicit look at implicit learning: an interrogative review for sport coaching research and practice. Sports Coach Rev. (2023) 12(1):1–22. 10.1080/21640629.2023.2179300

[14] Garnaas HB van den Tillaar R. Comparison of effects of implicit versus explicit learning of a novel skill in young gymnastic athletes. Behav Sci. (2024) 14(9):798. 10.3390/bs14090798 39336013 PMC11429373

[15] Kal E Prosée R Winters M Van Der Kamp J. Does implicit motor learning lead to greater automatization of motor skills compared to explicit motor learning? A systematic review. PLoS One. (2018) 13(9):e0203591. 10.1371/journal.pone.0203591 30183763 PMC6124806

[16] Matsuura Y Motoya S Amemiya R Sakairi Y. An instructional strategy for gymnastics based on versatile kinesthesis experience and its effects on enjoyment and performance. Jpn J Phys Educ Health Sport Sci. (2018) 63(1):265–80. 10.5432/jjpehss.17059

[17] Verburgh L Scherder EJA Van Lange PAM Oosterlaan J. The key to success in elite athletes? Explicit and implicit motor learning in youth elite and non-elite soccer players. J Sports Sci. (2016) 34(18):1782–90. 10.1080/02640414.2015.1137344 26788666

[18] Sun R Merrill E Peterson T. From implicit skills to explicit knowledge: a bottom-up model of skill learning. Cogn Sci. (2001) 25(2):203–44. 10.1207/s15516709cog2502_2

[19] Sun R Slusarz P Terry C. The interaction of the explicit and the implicit in skill learning: a dual-process approach. Psychol Rev. (2005) 112(1):159–92. 10.1037/0033-295X.112.1.159 15631592

[20] Dishman RK Motl RW Saunders R Felton G Ward DS Dowda M Enjoyment mediates effects of a school-based physical-activity intervention. Med Sci Sports Exerc. (2005) 37(3):478–87. 10.1249/01.MSS.0000155391.62733.A7 15741848

[21] Csikszentmihalyi M. Beyond Boredom and Anxiety. San Francisco, CA: Josses-Bass (1975).

[22] Heutte J Fenouillet F Kaplan J Martin-Krumm C Bachelet R. The EduFlow model: a contribution toward the study of optimal learning environments. In: Harmat L, Andersen FØ, Ullén F, Wright J, Sadlo G, editors. Flow Experience: Empirical Research and Applications. Cham: Springer International Publishing AG (2016). p. 127–43.

[23] Palomäki J Tammi T Lehtonen N Seittenranta N Laakasuo M Abuhamdeh S The link between flow and performance is moderated by task experience. Comput Hum Behav. (2021) 124:106891. 10.1016/j.chb.2021.106891

[24] Wilson C Simpson SE Van Emmerik RE Hamill J. Coordination variability and skill development in expert triple jumpers. Sports Biomech. (2008) 7(1):2–9. 10.1080/14763140701682983 18341132

[25] Stokes PD Lai B Holtz D Rigsbee E Cherrick D. Effects of practice on variability, effects of variability on transfer. J Exp Psychol Hum Percept Perform. (2008) 34(3):640–59. 10.1037/0096-1523.34.3.640 18505329

[26] Kaneko A. Coaching in Artistic Gymnastics. Tokyo: Taishukan (1974).

[27] Kaneko A. Apparatus Gymnastics Instruction Method Series for Teachers 2: Mat Exercises. Tokyo: Taishukan (1982).

[28] Miki S Kato S Motomura K. Latest Physical Education Lesson Series: Designing Gymnastics Lessons in Junior High and High Schools. Tokyo: Taishukan (2006).

[29] Yagi T Sakairi Y. Subjective arousal and experience of flow during positive sports events. Jpn J Health Psychol. (2009) 22(1):24–32. 10.11560/jahp.22.1_24

[30] Deterding S Dixon D Khaled R Nacke L. From game design elements to gamefulness: defining “gamification”. Proceedings of the 15th International Academic MindTrek Conference: Envisioning Future media Environments (2011). p. 9–15.

[31] Hamari J Koivisto J Sarsa H. Does gamification work? A literature review of empirical studies on gamification. 2014 47th Hawaii International Conference on System Sciences (2014). p. 3025–34.

[32] Stott A Neustaedter C. Analysis of gamification in education. Technical Report 2013-0422-01 (2013). Available at: http://carmster.com/clab/uploads/Main/Stott-Gamification.pdf (Accessed March 21, 2025).

[33] Moxley S. Schema: the variability of practice hypothesis. J Mot Behav. (1979) 11(1):65–70. 10.1080/00222895.1979.10735173 15186973

[34] Schmidt RA. A schema theory of discrete motor skill learning. Psychol Rev. (1975) 82(4):225–60. 10.1037/h0076770

[35] Harbourne RT Stergiou N. Movement variability and the use of nonlinear tools: principles to guide physical therapist practice. Phys Ther. (2009) 89(3):267–82. 10.2522/ptj.20080130 19168711 PMC2652347

[36] Dal’Bello LR Izawa J. Computational role of exploration noise in error-based de novo motor learning. Neural Netw. (2022) 153:349–72. 10.1016/j.neunet.2022.06.011 35779444

[37] Abswoude FV. Implicit and explicit motor learning in children: an investigation into individual differences to promote tailor made motor learning in practice. Proefschrift-AIO (2019). Available at: https://repository.ubn.ru.nl/bitstream/handle/2066/207487/207487.pdf (Accessed March 21, 2025).

